# The impact of a self-development coaching programme on medical and dental students’ psychological health and academic performance: a randomised controlled trial

**DOI:** 10.1186/s12909-015-0412-4

**Published:** 2015-08-19

**Authors:** Khalid Aboalshamat, Xiang-Yu Hou, Esben Strodl

**Affiliations:** 1 Community Dentistry, Faculty of Dentistry, Umm Al-Qura University, Makkah, Saudi Arabia; 2 School of Public Health and Social Work, Queensland University of Technology, Brisbane, Australia; 3 School of Psychology and Counselling, Queensland University of Technology, Brisbane, Australia

## Abstract

**Background:**

Psychological distress is well-documented worldwide among medical and dental students. Few studies have assessed the impact of self-development coaching programs on the students’ psychological health. The aim of the study was to evaluate the effect of a self-development coaching programme on the psychological health and academic performance of preclinical medical and dental students at Umm Al-Qura University, Saudi Arabia.

**Methods:**

Four-hundred and twenty-two participants (n = 422, 20–22 years) fulfilled the study requirements and were invited into a parallel-randomised controlled trial that was partially blinded. Participants were stratified by faculty, gender, and academic year, and then randomised. A total of 156 students participated in the intervention group (IG) and 163 students participated in the control group (CG). The IG received the selfdevelopment programme, involving skills and strategies aimed to improve students’ psychological health and academic performance, through a two-day workshop. Meanwhile, the CG attended an active placebo programme focussing on theoretical information that was delivered through a five-hour workshop. Both programmes were conducted by the same presenter during Week 1 of the second semester of the 2012–2013 academic year. Data were gathered immediately before (T1), one week after (T2) and five weeks (T3) after the intervention. Psychological health was measured using the Depression Anxiety Stress Scale (DASS-21), the General Self-Efficacy (GSE), and the Satisfaction With Life Scale (SWLS). Academic performance was measured using students’ academic weighted grades (WG). Student cognitive and emotional perceptions of the intervention were measured using the Credibility/Expectancy Questionnaire (CEQ).

**Results:**

Data from 317 students, who completed the follow ups, were analysed across the three time periods (IG, n = 155; CG, n = 162). The baseline variables and demographic data of the IG and CG were not significantly different. The IG showed short-term significant reductions in depression and anxiety in compared to CG from T1 to T2. The short-term changes in stress, GSE and SWLS of the IG were not significantly different from those of the CG. While both groups showed a significant change on most of the psychological variables from T1 to T3, no significant differences were found between the groups in this period. In addition, no significant difference was found in WG between the IG and CG after the intervention. No harms relevant to the intervention were reported.

**Conclusion:**

The investigated self-development coaching programme showed only a short-term improvement on depression and anxiety compared with an active control. There was no effect of the intervention on academic performance.

**Trial registration:**

ACTRN12614000896673

## Background

Psychological health disturbances, including depression, anxiety and stress, are common and well-documented worldwide among dental and medical students [1–7]. Medical and dental students seem to have poorer psychological health than their peers in the general population [1, 8, 9]. The status of these students’ psychological health has also been manifested in terms of low levels of self-efficacy and low levels of satisfaction with life [10, 11]. University students’ poor psychological health is also of significant interest, as it may persist into their professional lives, affect patient safety [12] or lead the students to leave their health profession [13].

Commercial self-development coaching programmes are popular among the general population to enhance people’s psychological health [14]. Self-development coaching programmes are defined as ‘interactive, multidimensional human developmental process, mainly between non-clinical coachees and a trusted coach who has a number of characteristics to facilitate an individual’s life improvement, which could extend sub-sequentially to the organization, in fields valued by the coachees, using a combination of proven and unproven techniques and concepts’ [15]. In fact, self-development coaching programmes are similar to ‘life coaching’, which has a fledgling but growing scientific evidence base [15]. Only a few interventional studies have investigated the effectiveness of self-development programmes on psychological health. A quasi-experimental study involving medical students in Norway exhibited a significant reduction in stress after a 12-week self-development programme [16]. Another study focussing on the general population in Sweden found an improvement in quality of life after a one-week self-development programme [17]. These studies encourage the investigation of the effectiveness of such programmes, especially for medical and dental students, since only a few rigorously evaluated interventions have been conducted on such populations [18].

Given the popularity of self-development coaching programmes and the paucity of research examining the effectiveness of such programmes in improving psychological health, there is a need for further studies to test their effectiveness empirically. A pilot study was previously conducted in Saudi Arabia to investigate the effect of the self-development coaching programme on medical students [19]. The study found that depression, self-efficacy and satisfaction with life improved significantly after attending the programme. However, the pilot study involved a small sample size, only one follow-up wave and no control group.

As such, this study aimed to build upon our previous pilot study to examine the effectiveness of a self-development coaching programme in improving the psychological health and academic performance of preclinical medical and dental students in Saudi Arabia. Specifically, we sought to answer the following two questions: (1) Does the self-development coaching programme have a short and/or longer term effect on the students’ psychological health? (2) Does the programme affect students’ academic performance?

## Methods

### Study design and participants

This study used a parallel-grouped randomised control trial (RCT) design where the control group received a placebo intervention. Documenting this study was conducted following CONSORT guidelines. The target population was preclinical medical and dental students at Umm Al-Qura University (UQU), Makkah, Saudi Arabia in the 2012–2013 academic year. The students’ age range was 20–22 years. The preclinical medical and dental students study a traditional curriculum (lecture-based) with compulsory course unit structure, and they are assessed by essays, multiple-choice and objective structured clinical examination. The medical/dental programme is composed of one orientation year, two preclinical years (2^nd^–3^rd^) and three clinical years (4^th^–6^th^), followed by an internship year. Each academic year is composed of two terms with a summer vacation. Eligibility criteria were (a) being a medical or dental student; (b) being a second- or third-year student; and (c) studying at UQU. Exclusion criteria included students who (a) attended the interventional programme during the course of their academic study, (b) under psychological treatments or drugs regimen or (c) did not sign the study consent form.

A sample size of 130 (65 at each group) participants was needed to detect a difference between the two groups. A study power of 90 %, type I error of 5 %, minimal clinical difference of 4 points in any of the psychological health means, and an average standard deviation of 7, derived from a recent well-designed coaching RCT which used the Depression Anxiety Stress Scale (DASS-21) [20], were used in the sample size calculation. The resulting number (130) for the two groups was multiplied by 1.5 for the design effect (multiple follow-up), yielding a desired sample size of 196 in both groups. This number was again multiplied by 1.5 for the estimated non-response rate (50 %) and multiplied by 1.25 for estimated drop-out during the follow-up (20 %), with the result that 366 students needed to be approached.

### Setting

The study was advertised via large roll-up posters, and students were recruited in the first term via invitation envelopes which contained coloured flyers about the programmes, a study information sheet and the consent form. After receiving participants’ signed consent, participants were randomly allocated into the intervention group (IG) and control group (CG) by the principal investigator. Randomisation was achieved using a computer-generated random number list. The intervention was conducted during the first week of the second term. Students knew their assigned group one week before both programmes were conducted. However, the students and research assistants who managed the study protocol and data collection were blinded to the participants’ group allocation. Thus, the study was partially blinded. Students were assessed three times in the second term, as follows: Week 1 (T1), immediately before the programme was conducted; Week 2 (T2), a week after the programme; and Week 6 (T3), five weeks after the programme concluded. The questionnaires were disseminated and collected between students’ lecture breaks.

### Intervention

Students in the IG attended a self-development coaching programme titled “How to Be an Ultra Super Student” (HBUSS), while the control group received a normal lecture-type programme titled “Learning and Success in Health Faculties” (LSHF). Each programme was delivered as a live course in a large lecture theatre during students’ free time in Week 1 of the second term; participants in both groups were supplied with the appropriate programme booklet and audio CD. Due to cultural and religious considerations in Saudi Arabia, male and female seats were separated by a barrier along the theatre, but facing the coach, on the intervention days.

The HBUSS is a self-development coaching programme, which has been developed and run by the lead author, who is a self-development coach and trainer, since 2008 [19]. The contents were derived from the coach’s personal experiences with coaching and from reading and practicing self-development over a number of years. The programme aimed mainly to improve students’ academic performance and psychological health. It did not use psychological therapeutic approaches, but rather focussed on a series of skills and conceptual ideas about studying and coping with challenges during the academic journey.

On the other hand, the LSHF programme was developed by the first author for the purpose of this study only. It provided information about learning in health faculties and the factors leading to success according to a scientific literature review. It also briefly touched on the scientific research area. The information in the LSHF programme was taken from academic articles or books; however, it did not have a practical aim to improve students’ performance or psychology. Both programs were presented by the first author. The programmes’ modules, CD contents, approach and duration are detailed in Table 1.

**Table 1:** Comparison between the intervention and control programmes

	Interventional group programme	Control group programme
Programme name	‘How to Be an Ultra-Super Student’	‘Learning and Success in Health Faculties’
Course modules	(1) Unleash your inner power: information about self-efficacy and goals in life is discussed.	(1) Bloom’s taxonomy [37]: cognitive, affective and psychomotor learning levels.
	(2) Manage your time effectively: different models and tips to utilize studying time efficiently.	(2) Scientific literature on variables association with success in health faculties such as the language, income, etc., with no practical points.
	(3) The maximum usefulness of university lectures: different solutions to increase lecture time efficiency.	(3) Active learning potential use in health faculties.
	(4) How to study and memorise effectively: skills with exercises to memorise better.	(4) The importance of scientific research.
	(5) Dealing with exams: practical tips to deal with exam time.	
	(6) Religious teaching: Islamic teaching augments the previous skills and values in the Saudi religious and cultural context.	
Audio CD contents	(1) Twenty-four study-motivation audio files.	Twenty-four audio files reiterating the contents of the programme.
	(2) Short version of muscle relaxation and positive messages.	
	(3) Long version of muscle relaxation and positive messages.	
Approaches to conducting the programme	• Motivational vocal tone and body language.	• Normal, standard approach to presenting a lecture at university.
	• Success and Islamic stories (parables).	• Short questions to be answered individually or in groups.
	• Famous people and Islamic quotes (metaphor).	
	• Recontextualised ideals.	
	• Personification of some values.	
	• Movie clips.	
	• Direct interaction with the audience.	
	• Giving coachees the freedom to choose amongst the programme techniques which suit them.	
Duration	Two days (10-h programme), with multiple 10–40 min breaks.	One day (4-h programme), with multiple 10–40 min breaks.

Note: The information of the intervention group was replicated from the pilot study [19] with slight modification

While the presenter was careful to follow the intervention and active control group manuals, no independent assessment of treatment fidelity was performed. In addition, while the participants’ attendance was tracked, there was no assessment of completion of quizzes or assignments associated with the workshop material in order to assess the participants’ understanding of the material. This was not done as it is not a common practice for self-development coaching programs, in contrast to other formal training courses, and so would have altered the participants’ experience of a typical self-development coaching program.

### Assessment

Hard copies of the self-report questionnaire were used. Three aspects were assessed, as follows: (a) psychological health at T1, T2 and T3; (b) participants’ levels of belief in the effectiveness of the programme, considered both logically and emotionally at T1 and T2; and (c) students’ academic performance before and after the intervention.

Psychological health was measured using the DASS-21 [21, 22], General Self-Efficacy scale (GSE) [23], and Satisfaction With Life Scale (SWLS) [24]. The DASS-21 measured negative aspects of students’ psychological health, while GSE and the SWLS measured positive aspects of students’ psychological health. The DASS-21 is composed of 21 questions to assess depression, anxiety and stress subclasses, which are measured by the sum of the 7 corresponding questions. Each question can be answered from 0 “Did not apply to me at all” to 3 “Applied to me very much, or most of the time”. A high DASS-21 subclass score indicates unfavourable status. DASS-21 has excellent psychometric properties, with a Cronbach’s alpha of 0.82 to 0.90 for each subscale [25]. GSE is composed of 10 questions to measure self-efficacy, and each question can be answered from 1 “Not at all true” to 4 “Exactly true”. GSE has a Cronbach’s alpha of .86 among 25 nations [26]. Finally, the SWLS is composed of five questions to measure life satisfaction, and each question can be answered from 7 “Strongly agree” to 1 “strongly disagree”. SWLS has a Cronbach’s alpha of .87 [27, 28]. High SWLS or GSE sum-scores indicate high satisfaction or self-efficacy.

In addition, the Credibility and Expectancy Questionnaire (CEQ) [29] was used to investigate participants’ perception levels of the programme’s success, both logically (credibility) and emotionally (expectancy). This is a 6-item scale, with some answers ranging from 1 “Not at all confident” to 9 “Very confident”, and others ranging from 0–100 %. The Cronbach’s alpha of the CEQ is .85 [29]. The validated Arabic versions of the DASS-21 and GSE were used [26, 30], while the SWLS and CEQ were face and content validated and translated into Arabic in the pilot study [19] using World Health Organization (WHO) translation guidelines [31].

Academic performance was measured by students’ weighted grades (WG), in the first term before the intervention and at the end of the second term, four months after the intervention. WG were measured according to the following equation:

$$ \mathrm{Weighted}\ \mathrm{grade}\ \mathrm{percentage}\ \left(\mathrm{W}\mathrm{G}\right)={\displaystyle \sum }\ \frac{\left(\mathrm{each}\ {\mathrm{unit}}^{\hbox{'}}\mathrm{s}\ \mathrm{grade}\mathrm{s}\ *\ {\mathrm{unit}}^{\hbox{'}}\mathrm{s}\ \mathrm{credit}\ \mathrm{hours}\right)}{\left(\ \mathrm{total}\ \mathrm{units}\ \mathrm{credit}\ \mathrm{hours}\right)}*10 $$

Student’s grades were obtained from faculties’ administrative offices after receiving the students’ approval. Demographic data included faculty, academic year, gender, family income, marital status and nationality. All identifying information was destroyed after data completion and the data were treated anonymously.

### Incentives and ethical considerations

Participation in the study was voluntary. All participants received the interventional or placebo programmes without charge. Students received two certificates of appreciation, one upon attending the designated programme, and one after completing all the follow-ups. All attending students were entered in three random prize draws for 50 Saudi Riyal (13.33 U.S dollar) vouchers in each programme.

The study was approved by the Queensland University of Technology ethical committee. As an institutional ethics board had not been formally established at UQU, formal approvals were obtained from the medical and dental faculties at UQU, in addition to the students’ signed consent.

### Randomisation

Participants were stratified by faculty, gender, and academic year, and then randomised into the IG and CG using computer-generated random number lists by the principal investigator. Stratified randomisation was conducted mainly to overcome the unbalanced medical/dental student ratio. Neither students nor research assistants were aware of which students were allocated to the IG and CG until the first day of the second term.

### Data analysis

SPSS software package version 21 was used to assist in data analysis. Chi-square and Fisher’s exact tests were employed to test the demographic variable differences between the groups. A *t*-test was used to compare the baselines of the DASS-21 subgroups, GSE, SWLS and CEQ. After splitting data by group, repeated measures analysis of variance (rANOVA) was used to test the difference between T1 to T2, T2 toT3 and T2 to T3 for the IG and CG. Factorial rANOVA was used to analyse the differences in all the outcome variables between IG and CG. Bonferroni correction was used for the rANOVA post hoc test.

## Results

**Fig. 1:**
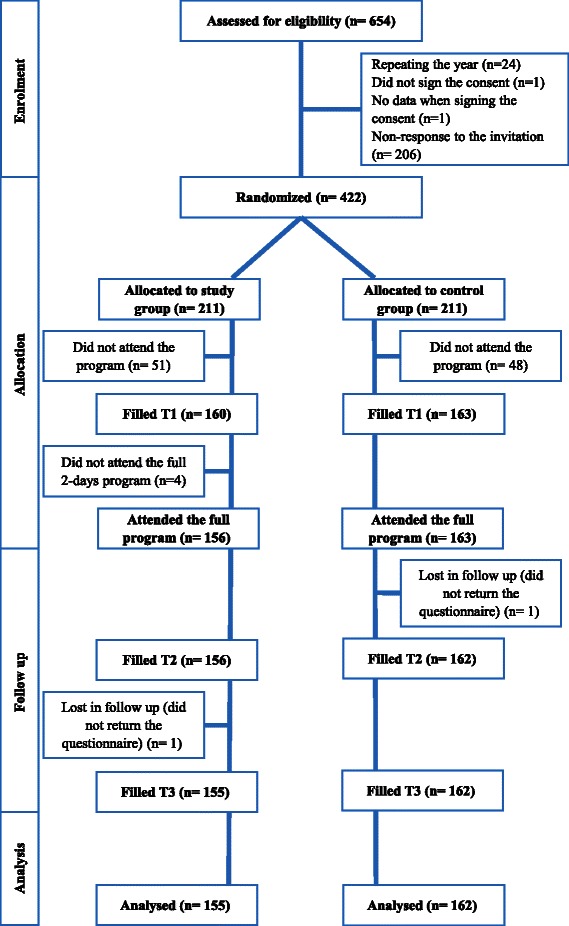
Flow of participants through the study. Flow of participants through the study

The participants’ flow chart is detailed in Fig. 1. Among all the students, 422 signed the consent to participate in the study and became eligible to participate, resulting in an initial 64.25 % response rate. Of the students, 319 attended the programmes, and only two were lost to follow-ups, resulting ultimately in a 25.88 % drop-out rate. Students’ demographic data are displayed in Table 2. Using the Chi-square and Fisher’s exact tests, there was no significant difference in the demographic variables (Table 2).

**Table 2:** Description of the demographic data of the participants from medical and dental students at preclinical years at UQU

	Interventional group	Control group	Total
	No. (%) *n* = 155	No. (%) *n* = 162	No. (%) *n* = 317
Gender			
Male	68 (43.9)	76 (46.9)	144 (45.4)
Female	87 (56.1)	86 (53.1)	173 (54.6)
Marital status			
Single	153 (98.7)	157 (96.9)	310 (97.8)
Married	2 (1.3)	5 (3.1)	7 (2.2)
Family income			
Low^a^	46 (29.7)	61 (37.7)	107 (33.8)
High^b^	109 (70.3)	101 (62.3)	210 (66.2)
Nationality			
Saudi	151 (97.4)	160 (98.8)	311 (98.1)
Non-Saudi	4 (2.6)	2 (1.2)	6 (1.9)
Academic year			
2nd year	75 (48.4)	79 (48.8)	154 (48.6)
3rd year	80 (51.6)	83 (51.2)	163 (51.4)
Faculty			
Medicine	126 (81.3)	133 (82.1)	259 (81.7)
Dentistry	29 (18.7)	29 (17.9)	58 (18.3)

^a^Low family income: less than 10,000 Saudi Riyal/ month (2,666.67 U.S dollar)

^b^High family income: more than 10,000 Saudi Riyal/ month (2,666.67 U.S dollar)

Note: Using Chi-square and Fisher’s exact test for cells count less than 5, there was no significant difference in the demographic variables between the two groups

Table 3 shows the means and standard deviations for all measured variables for IG and CG. Using *t*-test, the psychological variables, CEQ and WG’s baseline measures were not significantly different between the IG and CG (Table 3).

**Table 3:** The mean scores for depression, anxiety, stress, GSE, the SWLS, credibility, expectancy and WG at T1, T2, T3 for the IG and CG, and the results of the rANOVA after data were split by group

		T1 *M (SD)*	T2 *M (SD)*	T3 *M (SD)*
Depression	IG	12.79 (9.42)^ab^	7.08 (7.04)^c^	9.28 (8.6)
	CG	12.32 (9.31)^ab^	8.76 (7.76)	8.88 (8.27)
Anxiety	IG	11.36 (9.15)^ab^	5.99 (6.20)^c^	7.46 (8.1)
	CG	9.97 (8.48)^ab^	6.79 (6.88)	7.31 (6.95)
Stress	IG	16.81 (9.94)^ab^	11.03 (7.43)	11.24 (8.56)
	CG	16.06 (9.02)^ab^	11.57 (8.71)	12.12 (8.66)
GSE	IG	27.45 (4.71)^ab^	28.49 (5.25)	28.48 (5.69)
	CG	27.17 (4.20)	27.70 (4.38)	27.50 (5.04)
SWLS	IG	24.13 (6.61)^ab^	25.81 (6.43)	25.42 (6.31)
	CG	24.31 (6.11)^a^	25.35 (6.38)	24.67 (6.63)
Credibility	IG	23.29 (5.91)^a^	26.06 (6.03)	-
	CG	23.00 (5.08)^a^	21.02 (7.00)	-
Expectancy	IG	23.46 (6.34)^a^	24.63 (6.61)	-
	CG	22.28 (5.76)^a^	19.52 (7.85)	-
WG^d^	IG	81.1 (8.42)^a^	83.55 (7.22)	-
	CG	80.26 (10.31)^a^	82.56 (6.91)	-

Abbreviation: *IG* interventional group; *CG* control group; *T1* before the intervention at week 1; *T2* one week after the intervention at week-2; *T3* five weeks after the intervention at week 6; *M* mean; *SD* standard deviation; *GSE* General Self-Efficacy; *SWLS* Satisfaction With Life Scale

^a^p-value < .05 for rANOVA post hoc test for T1-T2

^b^p-value < .05 for rANOVA post hoc test for T1-T3

^c^p-value < .05 for rANOVA post hoc test for T2-T3

^d^WG in column T1 = students’ weighted grades before the intervention, and in column T2, after the intervention, for simplicity in data presentation

All the variables had acceptable levels of skewness (−1.55 to 1.38), and kurtosis ranged from (−.3 to 1.88), except for WG after the intervention with kurtosis of 3.74. Thus, parametric tests were used, even for WG because the chosen tests are robust. Sensitivity analysis using non-parametric tests (Mann–Whitney U, Friedman, and Wilcoxon tests) showed the same reported significance for the parametric tests. The Cronbach’s alpha for the measures of depression, anxiety, stress, GSE, and SWLS were .86, .83, .84, .84, and .82, respectively.

**Fig. 2:**
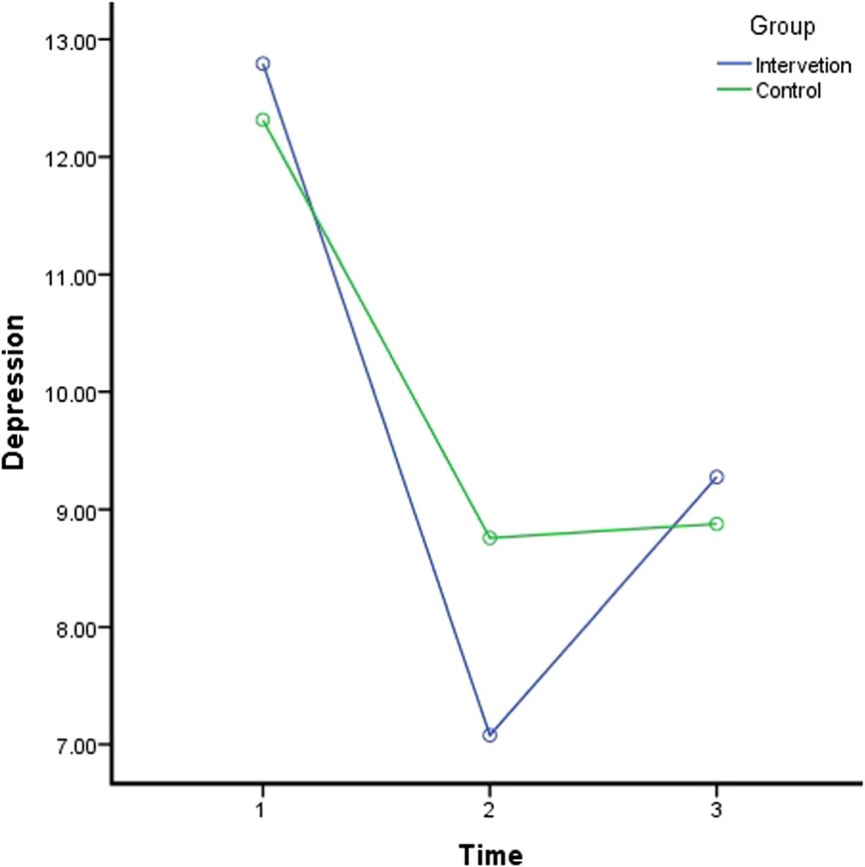
Depression for groups across time. Depression for groups across time

**Fig. 3:**
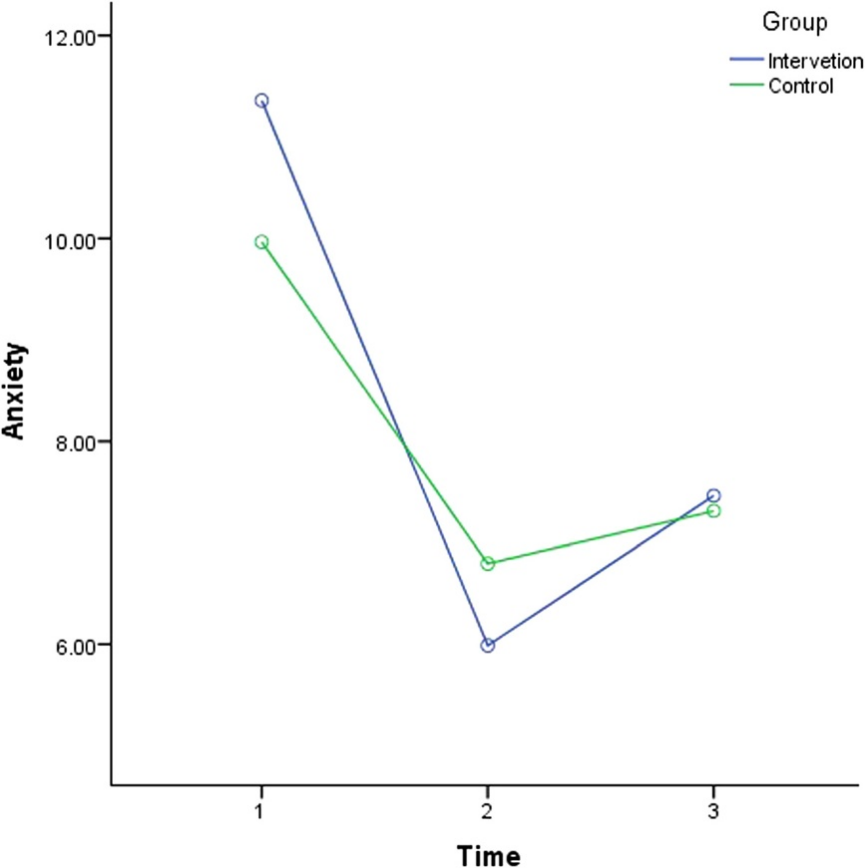
Anxiety for groups across time. Anxiety for groups across time

**Fig. 4:**
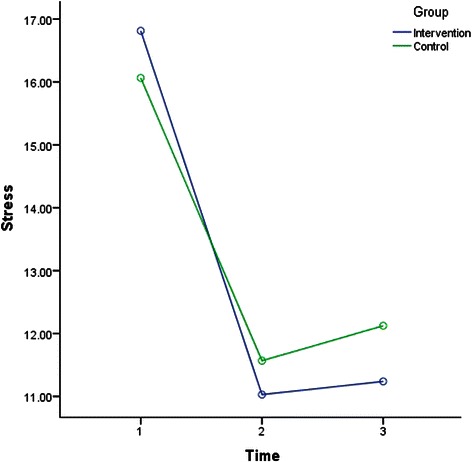
Stress for groups across time. Stress for groups across time

**Fig. 5:**
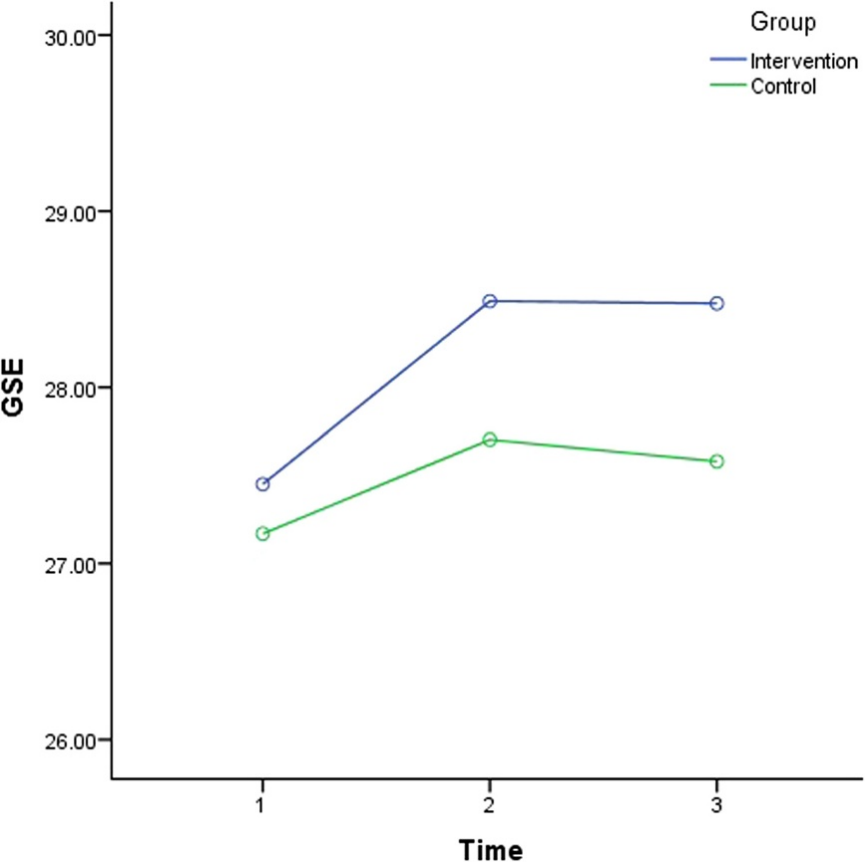
GSE for groups across time. GSE for groups across time

**Fig. 6:**
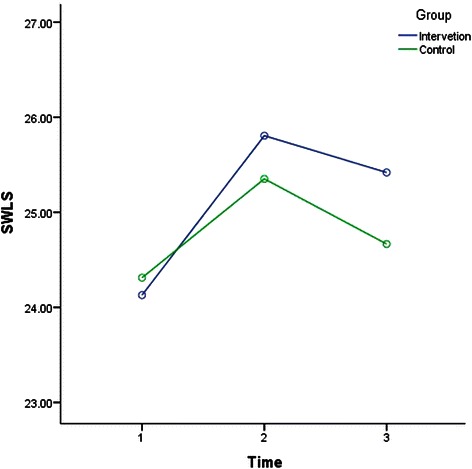
SWLS for groups across time. SWLS for groups across time

The results of rANOVA after splitting the data to analyse IG and CG across time (T1-T2, T1-T3, and T2-T3) were included in Table 3. Factorial rANOVA results were detailed in Table 4. Table 4 also shows the results of the post hoc test to compare between the interaction of the groups and different time points. Depression, anxiety, stress, SWLS, GSE for IG and CG measures across time are illustrated in Figs. 2, 3, 4, 5 and 6.

**Table 4:** Factorial rANOVA and post hoc test results for the interaction of groups and depression, anxiety, stress, GSE, the SWLS, credibility, and expectancy

	Within subject effect *df(F), p*	Between subject effect *df(F), p*	Post hoc test Time *group *df(F), p*
Depression	1.91(4.22), *p* = .017	1.91(4.22), *p* = .017	
T1-T2 *group			1(6.33), *p* = .012
T2-T3 *group			1(7.64), *p* = .006
T1-T3 *group			1(0.01), *p* = .934
Anxiety	1.83(66.14), *p* < .001	1.83(4.03), *p* = .021	
T1-T2 *group			1(7.96), *p* = .005
T2-T3 *group			1(2.08), *p* = .15
T1-T3 *group			1(2.03), p = .156
Stress	1.91(78.59), *p* < .001	1.91(1.77), *p* = .172	
T1-T2 *group			1(2), *p* = .158
T2-T3 *group			1(0.18), *p* = .676
T1-T3 *group			1(2.68), *p* = .103
GSE	1.93(7.23), *p* = .001	1.93(1.02), *p* = .358	
T1-T2 *group			1(1.4), *p* = .238
T2-T3 *group			1(0.06), *p* = .805
T1-T3 *group			1(1.52), *p* = .218
SWLS	1.91(16.3), *p* < .001	1.91(1.99), *p* = .14	
T1-T2 *group			1(1.97), *p* = .161
T2-T3 *group			1(0.44), *p* = .51
T1-T3 *group			1(3.14), *p* = .077
Credibility	1(1.23), *p* = .269	1(45.17), *p* < .001	
T1-T2 *group			1(45.17), *p* < .001
Expectancy	1(4.68), *p* = .031	1(28.14), *p* < .001	
T1-T2 *group			1(28.14), *p* < .001
WG	1(86.06), *p* < .001	1(1.55), *p* = .214	
T1-T2 *group^a^			1(1.55), *p* = .214

Abbreviation: *T1* before the intervention at week 1; *T2* one week after the intervention at week-2; *T3* five weeks after the intervention at week 6; *M* mean; *SD* standard deviation; *df* degrees of freedom; *p* p-value; *GSE* General Self-Efficacy; *SWLS* Satisfaction With Life Scale

* refers to statistical interaction

^a^For WG row, T1 = students’ weighted grades before the intervention, and in column T2, after the intervention, for simplicity in data presentation

In general, Table 4 indicates that the within-subject effect of time was significant for depression, anxiety, stress, GSE, SWLS, expectancy, WG, but not credibility. Moreover, the interactions between time and group were only significant in terms of depression, anxiety, credibility, and expectancy. According to Table 3, depression, anxiety and stress in IG and CG improved (decreased) significantly from T1 to T2 (one week period). However, the post hoc test in Table 4 indicates that the improvement from T1 to T2 was significantly more in IG than CG. Table 3 shows that depression, anxiety and stress were significantly higher at T3 in compared to T1 (five weeks apart) in both groups, but Table 4 indicates that the improvement from T1 to T3 was not significantly different between IG and CG.

Also, Table 3 shows that GSE level in IG improved (increased) significantly from T1 to T2 (one week period), while SWLS improved in both groups significantly in the same period. However, the post hoc test in Table 4 indicates that the improvement within GSE and SWLS from T1 to T2 was not significantly different between IG and CG. Table 3 also shows that GSE and SWLS were significantly higher in T3 in compared to T1 (five weeks apart) in IG only, but Table 4 indicates that the improvement from T1 to T3 was also not significantly different between IG and CG.

Tables 3 and 4 shows that both credibility and expectancy increased significantly in the IG, whereas they decreased significantly in the CG after the intervention. Finally, the results in Tables 3 and 4 show that WG improved after the programmes in both the IG and CG. However, this improvement was not significantly different between IG and CG. Among all of the psychological and performance outcome variables, no harm was detected or reported.

## Discussion

This study aimed to explore the impact of a self-development coaching program, in comparison with an active control, upon the psychological health and academic performance of medical and dental students in Saudi Arabia. The results of the study indicate that the intervention had only a significant short-term (one week) effect on depression and anxiety on the students compared with the control group. However the intervention appeared to have no long-term (5 weeks) effect on the students’ psychological health or academic performance compared to those in the CG.

### The short term effect of the program

In terms of the short-term effect of the intervention on the students’ psychological health, depression, anxiety and stress means were reduced significantly in both groups. However, IG exhibited a greater reduction in depression and anxiety only after one week. This improvement in depression, anxiety and stress were considerable, given that the reported means reduced at T1 from means classified as mild and moderate into normal means according to the DASS-21 scoring guide [21, 22]. In addition, GSE was increased significantly in IG compared with the active control group, while SWLS was increased significantly in both groups. However, the short term improvement was not significantly different between IG and CG in GSE or SWLS.

### The long term effect of the program

Although the intervention group displayed a greater reduction in depression and anxiety compared with the active control group from T1 to T2, this was not maintained from T1 to T3. This appeared to be due to a significant increase in the levels of depression and anxiety from T2 to T3 in the intervention group.

This indicates that the long term improvement of depression and anxiety may not be maintained in IG. However a longer time frame is required to have greater confidence with such a conclusion. Both general self-efficacy and satisfaction with life appeared to improve with the intervention and this was maintained from T2 to T3, while weighted grades improved from T1 to T2. However there were no significant differences between the intervention and active control group indicating that both interventions may have had an impact on these variables.

Credibility and expectancy were similar in the IG and CG at the baseline, indicating that participants did not have prior cognitive or emotional biased perceptions of the HBUSS programme. Nevertheless, IG participants showed an increase in credibility and expectancy levels, while those of the CG had decreased. This indicated that students were able to identify the beneficial programme.

The observed effects are notable given that medical students are more likely to be distressed in the middle of the academic term, when exams take place and more assignments are due, compared to the beginning of the term [32]. The results support the view that such interventions can be useful if conducted towards the middle or the end of the academic year, when students’ psychological health is more likely to deteriorate [32, 33].

It is also interesting to note that even the students in the CG had a favourable psychological improvement during the follow-ups compared to the baseline. This suggests that either a placebo effect was present with the active control group, or else some aspect of the active control group had an impact upon the participants. This point should be addressed in further studies’ by adding a waitlist control group.

### Our results compared to the literature

When Holm et al. investigated the effect of a self-development intervention on third-year medical students in Norway, they found a significant improvement in students’ stress and psychological health three months after the intervention [16]. This contradicted our findings in terms of both an effect upon stress, as well as maintenance of improvements of depression or anxiety in the intervention group. This can be explained by several factors, including the length of Holm et al.’s intervention, which was three months (1.5 h/week) in contrast to our compact two-day programme, which may suggest that programs delivered in shorter segments over longer periods may be more helpful for students than programs that deliver a large amount of content over a relatively short period. In addition, the different content of both programmes may be a contributing factor. Another possible explanation is that in Holm et al.’s study, students were able to choose to participate in the self-development group, increasing the chance of selection bias, while students in our study were allocated randomly. Furthermore, the programme in Holm et al.’s study was conducted by a psychotherapist, while that in our study was not. Finally, different scales were used, which might have resulted in this difference.

Fernros et al.’s study also showed a significant improvement in health-related quality of life over a 6 month period, following a one-week self-development programme, compared with a control group, in a sample from the general population [17]. Fernros et al.’s intervention was provided by a self-development coach and trainer who had been conducting this programme for many years, which is similar to our intervention. However the differences in findings might again be attributed to a number of factors. First, once again there were differences in dosage, with the Fernros et al. intervention involving 14 h of contact a day over a one-week period. Other explanations include the different contents of Fernros et al.’s intervention and the likelihood of selection bias, as all the participants were self-selected. Finally, it is more likely that participants who paid for the programme (3,055 euro) would perceive an improvement, as the cost might influence a placebo effect [34].

Two interventional studies that used life coaching as an intervention for university students [35, 36] also reported a significant reduction in depression, anxiety and stress after short-term follow-up using the DASS-21. Also similar to our study, the authors did not find an effect on students’ academic performance [35]. These findings are in line with our results, with the exception that our study did not find a difference between the intervention and control groups on change in stress. These studies by Grant et al., did involve small sample sizes (<25) so it is possible that interventions presented in smaller groups than those used in our study may have a bigger impact upon the reduction of stress in university students. Another explanation is that life coaching in these studies was conducted by professional psychologists who depended mainly on facilitation processes to help the coachees to achieve pre-settled goals. This different approach might be more effective on stress than our intervention. Nevertheless, this similarity indicates that life coaching might have comparable effects on self-development coaching programmes.

### Strengths and limitations

This study had several strengths, including the partially blinded RCT design, the placebo intervention (the first according to our knowledge among coaching interventions), the validated instruments used, the large sample size and the relatively low percentage of drop-out in such a study design. In fact, this study is considered the first intervention in the Middle East and the Arab world attempting to improve psychological health. However, a number of limitations should be acknowledged. The first author was the coach for the intervention and responsible for the randomisation. The LSHF programme duration (one-day) was not matched with the HBUSS programme (two-days). However, this was an attempted to reduce the anticipated students’ drop-out rate in CG in a second day. Also, there was a level of complexity in attributing causes and effects; for example, it is hard to identify the influential part among the program’s module or CD contents. Nevertheless, self-development programmes are usually given as one package of multiple modules to help the coachees with different issues.

Furthermore, the difference in original admission numbers between medical and dental faculties led to an unbalanced medical to dental students’ ratio. In addition, the study’s results cannot be generalised to all self-development coaching programmes, as they differ according to contents and presenters; neither can they be generalised to medical and dental students throughout Saudi Arabia. More importantly, longer follow-up periods and a waitlist control group were needed, and a better understanding of outcomes. Such points should be considered in future studies’ protocols.

## Conclusion

The self-development coaching programme ‘How to Be an Ultra Super Student’ seems to be a promising way to improve medical and dental students’ psychological health. The programme had only a short-term effect on some of the negative aspects of psychological health. However, no effect was shown on positive aspects or the students’ academic performance. The effect of the programme seems to be limited at the moment; however, given the importance of finding successful interventions for improving psychological health and academic performance in university students in Arab countries, further research building upon this study is recommended. Such research should explore the impact of changing aspects of the current intervention (such as the content, duration or delivery format) upon the improvement of medical and dental students’ psychological health and academic performance.

### Trial registration and protocol

The full protocol of the study can be retrieved by contacting the authors. The trial registration number is ACTRN12614000896673 at the Australian New Zealand Clinical Trials Registry.

## Abbreviations

CEQ: Credibility/expectancy questionnaire
DASS-21: Depression anxiety stress scale
GSE: General self-efficacy scale
HBUSS: How to be an ultra super student
IG: Interventional group
LSHF: Learning and success in health faculties
rANOVA: Repeated measures analysis of variance
RCT: Randomised control trial
SG: Study group
SWLS: Satisfaction with life scale
T1: Immediately before the programme conduction
T2: A week after programme conduction
T3: Five weeks after the programme conduction
U.S: United States of America
UQU: Umm al-qura university
WG: Students’ academic weighted grades
